# One-Step Fabrication of Pyranine Modified- Reduced Graphene Oxide with Ultrafast and Ultrahigh Humidity Response

**DOI:** 10.1038/s41598-017-02983-8

**Published:** 2017-06-02

**Authors:** Zhuo Chen, Yao Wang, Ying Shang, Ahmad Umar, Peng Xie, Qi Qi, Guofu Zhou

**Affiliations:** 1 Key Laboratory of Bio-Inspired Smart Interfacial Science and Technology of Ministry of Education, School of Chemistry and Environment, Beihang University, Beijing, 100191 P. R. China; 2 Department of Chemistry, Faculty of Science and Arts and Promising Centre for Sensors and Electronic Devices (PCSED), Najran University, Najran, 11001 Saudi Arabia; 3 Gas and Humidity Sensing Department, Beijing Elite Tech Co., Beijing, 100850 P. R. China; 4 Institute of Electronic Paper Displays, South China Academy of Advanced Optoelectronics, South China Normal University, Guangzhou, 510006 P. R. China

## Abstract

A facile one-step supramolecular assembly method is adopted to modify reduced graphene oxide (rGO) with functional organic molecule pyranine for achieving comprehensive humidity sensing performance. The fabricated humidity sensor based on pyranine modified-reduced graphene oxide (Pyr-rGO) exhibits excellent sensing performance with ultrafast (<2 s) and ultrahigh response of I_L_/I_H_ = 6000 as relative humidity (RH) consecutively changes between 11% and 95%; small hysteresis of 8% RH; reliable repeatability and stability. In addition, a detailed mechanism analysis is performed to investigate the difference in water adsorption and ions transfer under various RH levels. Notably, the one-step supramolecular assembly method to prepare Pyr-rGO provides a new insight into developing novel functional humidity sensing materials with enhanced device performance.

## Introduction

Humidity sensors have increasingly aroused interests due to the wide application in various realms, such as the indoor humidity measurement, industrial and agricultural environmental monitoring^1–3^. So far, several effective methods are proposed to fabricate humidity sensors while it still remains a challenge to make the fabrication more facile. For the whole sensor fabricating process, the selection of sensing materials is the most critical stage. Great efforts have been made to investigate the different sensing characteristics of various materials, including ceramics^4, 5^, metal oxide^6^, composites and organic polymers^7–9^. Nevertheless, it is hard to simultaneously realize the comprehensive humidity sensing properties such as rapid & high response, small hysteresis and reliable stability^10^. For instance, the ceramics materials possess excellent physical and chemical stability but the practical application is restricted since the need of auxiliary heat cleaning process. Thus, it is of great importance to explore a superior humidity sensing material which can endeavor more comprehensive properties.

Graphene, a fascinating material with perfect 2D atom-thin structure, large specific surface areas and excellent electronic properties, has been considered as a promising candidate for extensive applications including humidity sensing^11^. Several methods have been explored to prepare graphene such as mechanical exfoliation^12^, chemical vapor deposition^13, 14^, and epitaxial growth^15^, but the most effective approach should be the chemical reduction mainly because the potential for the relatively low-cost and large-scale fabrication from the view of practical application^16^. Unfortunately, the pristine reduced graphene oxide (rGO) without proper modification is not satisfying when applied as sensors, exhibiting slow response and low sensitivity towards gas or humidity changes^17, 18^. Moreover, the rGO nanosheets tend to irreversibly aggregate during the chemical reduction process, limiting the further devices fabrication. Hence, it is necessary to modify rGO with appropriate method.

Covalent chemical modification of rGO is widely used and indeed the sensing properties can be enhanced prominently, but usually a complex reaction process is required and the instinct structure and electrical properties of rGO will be damaged to some degree. To realize the mild and effective modification of rGO, a facile supramolecular assembly modification based on non-covalent interactions such as hydrogen bonding^19^, electrostatic forces^20^, and π-π stacking^21^, is an alternative approach which will maximize the remain of inherent properties of rGO^22^. In addition, supramolecular assembly modification allows more possibilities for functional “guests” such as inorganic ions, organic molecules or nanoparticles^17, 18^, which can be specific to enhance the dispersibility or humidity sensing performance of the as-prepared material.

In this work, we present a facile one-step supramolecular assembly method to modify rGO with functional organic molecule pyranine to form pyranine modified- reduced graphene oxide (Pyr-rGO) for achieving high performance humidity sensing materials. The as-prepared sensors exhibit excellent comprehensive humidity sensing properties including ultrafast (<2 s) and ultrahigh (I_L_/I_H_ = 6000) response. In addition, the humidity sensing mechanism under different RH levels has also been investigated in detail. It is worthy to mention that the one-step supramolecular assembly method has the potential for facilely preparing the novel functional materials with enhanced humidity sensing performance.

## Results

### The Supramolecular Assembly in Humidity Sensing Materials Preparation

It is well known that rGO sheets tend to aggregate due to the strong π-π interaction and hydrophobic surface, which is not desirable as humidity sensing materials. Thus, it is necessary to modify graphene by a mild approach to achieve well-dispersibility without damaging the instinct structure and electrical properties. Herein, a supramolecular assembly was introduced to non-covalently modify the graphene with aromatic molecules which enhanced the dispersibility of rGO in aqueous solution and adsorption of water molecules in the following humidity sensing applications. For this, a well-known organic aromatic molecule was selected, *i.e*. pyranine with a pyrene ring decorated with hydrophilic sulfonic groups.

**Figure 1:**
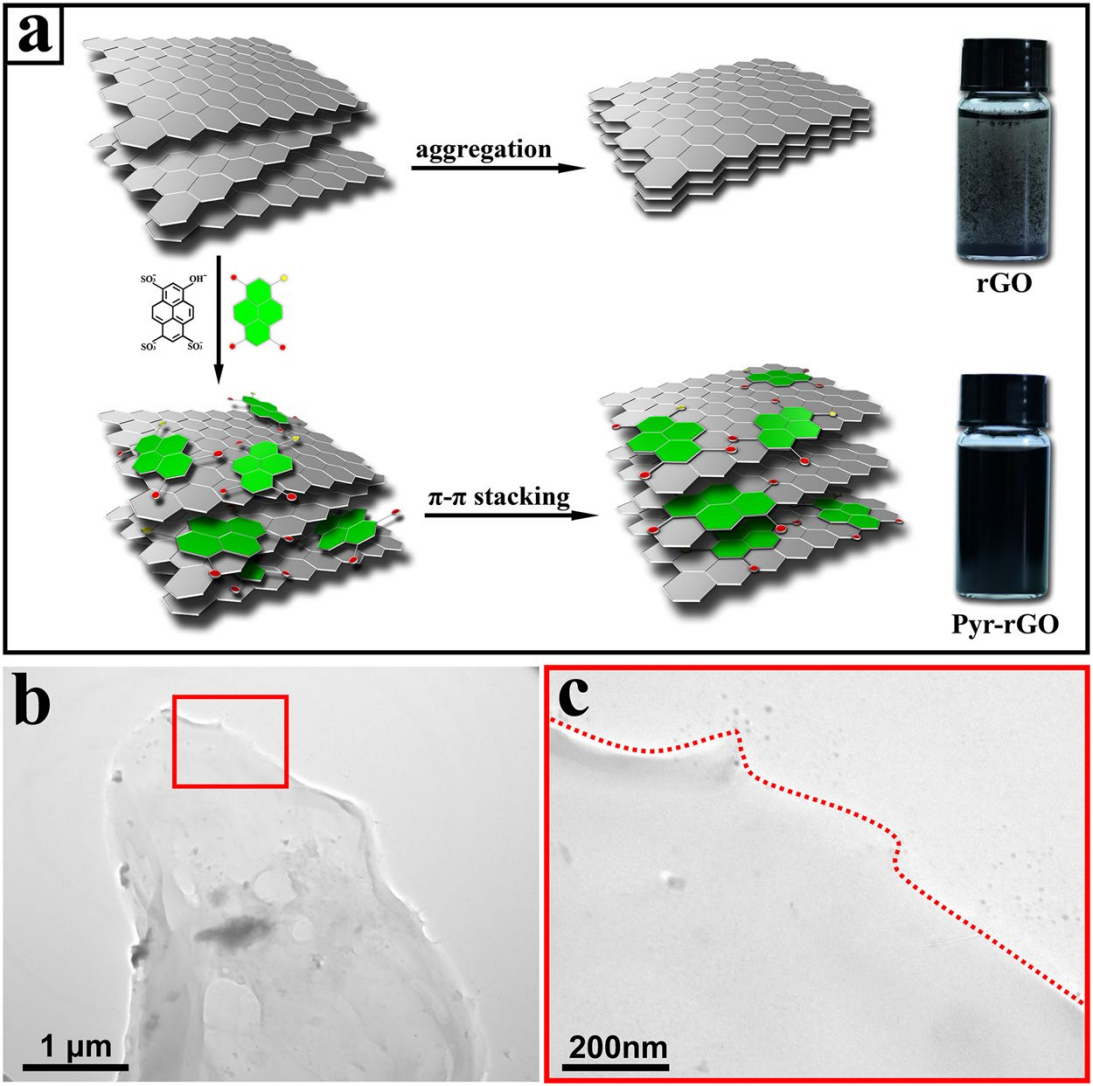
(**a**) The schematic of the aggregation by stacked rGO sheets and the supramolecular assembly of Pyr-rGO sheets with corresponding physical images in dispersion. (**b**) Low- and (**c**) high-magnification TEM image of the ultrathin Pyr-rGO film.

Figure 1a shows the physical images of rGO and Pyr-rGO dispersion with the same concentration of 1 mg/mL after static placement for a week. It is clearly shown that the rGO dispersion has aggregated while Pyr-rGO possesses stable aqueous dispersion with homogeneous dark color. The enhancement in dispersibility and stability of Pyr-rGO dispersion can be attributed to the pyranine molecules by supramolecular assembly. The large planar aromatic structure of pyranine can anchor themselves onto hydrophobic surface of rGO sheets via strong π-π interaction, and the hydrophilic sulfonic groups contribute a lot to the well-dispersibility (Fig. 1a). Moreover, the pyranine molecules act as superior stabilizer due to the electronegative sulfonic groups which maintain interlaminar static-repulsion forces and thus efficiently prevent the negative charged Pyr-rGO sheets from aggregation^23, 24^. The TEM images show the morphological features of Pyr-rGO sheets (Fig. 1b and c). At low magnification, a typical large-area and well-spread film is observed while the edge is difficult to distinguish even at high magnification, indicating that the film is ultrathin. In contrast, the TEM image shows rGO sheets have stacked together and form thick clusters (Supplementary Figure S1). It is worth mentioning that there is no covalent bonding between pyranine and graphene in the supramolecular assembly because the π-π interaction is a physical phenomenon driven by the electrostatic force rather than chemical reaction^25^, *i.e*. the modification would not disrupt the structure and electronic conjugation of graphene^26^.

### The Characterization

**Figure 2:**
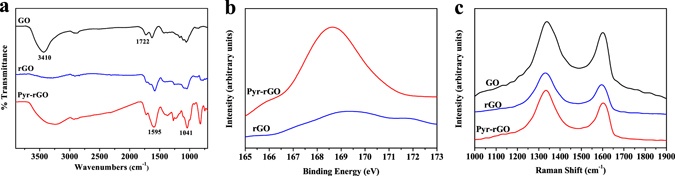
Typical (**a**) FTIR spectra of GO, rGO and Pyr-rGO. (**b**) XPS spectra of the Pyr-rGO and rGO, and (**c**) Raman spectra of the GO, rGO and Pyr-rGO.

Figure 2a shows the Fourier transform infrared (FTIR) spectroscopy analysis of GO, rGO and Pyr-rGO. Two obvious peaks at 3410 cm^−1^ and 1722 cm^−1^ assigned to hydroxyl and carboxyl groups respectively were observed in GO spectra. The peak at 3410 cm^−1^ had an attenuation in rGO spectra, and the attenuation also occurred at 1722 cm^−1^ in both rGO and Pyr-rGO spectra, suggesting that lots of oxygen-containing functional groups have been reduced by hydrazine hydrate. Notably, the peak at 3410 cm^−1^ reappeared in the spectra of Pyr-rGO, which should be attributed to the hydroxyl groups of pyranine molecule, and also the characteristic S=O peak around 1041 cm^−1^ and aromatic ring absorption peak at 1595 cm^−1^ appeared in Pyr-rGO spectra, which kept accordance with the spectra of pure pyranine powders as shown in Supplementary Figure S2, proving the successful assembly of pyranine molecules with rGO. XPS analysis was also conducted to investigate the surface properties and shown in Fig. 2b. Compared with rGO, Pyr-rGO spectrum appeared a prominent peak at 168.58 eV corresponds to S2p, suggesting the successful assembly of pyranine molecules on rGO, which was in accordance with the above FTIR analysis. The XPS peak table of Pyr-rGO was shown in Supplementary Table S1.

Raman spectroscopy was used to analyze the structural changes during the supramolecular assembly and reduction process. As indicated in Fig. 2c, the GO, rGO and Pyr-rGO spectrums all displayed two prominent Raman-active peaks at 1330 cm^−1^ and 1590 cm^−1^ assigned to the D- and G-band respectively. The rGO spectra exhibited the increased intensity ratio of the D-band to G-band (I_D_/I_G_ = 1.34) compared to GO (I_D_/I_G_ = 1.15), suggesting that the reduction process enhanced the defect content or edge area^27, 28^. Interestingly, Pyr-rGO showed the same I_D_/I_G_ value as rGO, proving that the supramolecular assembly modification is a mild approach without causing further disruption of rGO structure.

### Humidity Sensing Performance of Pyr-rGO

**Figure 3:**
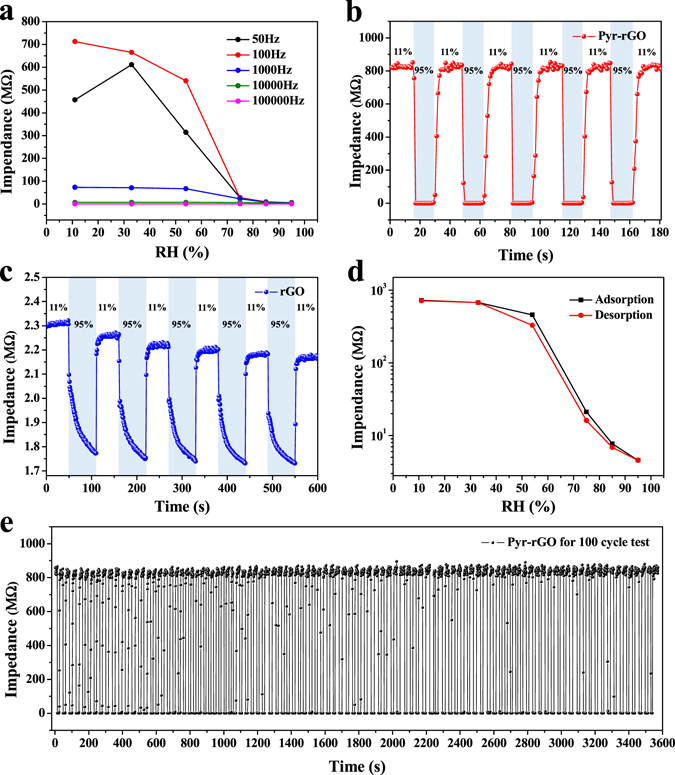
(**a**) The impedance curves of Pyr-rGO based humidity sensors measured at different frequencies under different RH levels. (**b**) The five-cycle response-recovery curve of Pyr-rGO and (**c**) rGO based humidity sensors measured at 100 Hz as RH level alternately changes between 11% and 95%. (**d**) Humidity hysteresis curve of Pyr-rGO based humidity sensors as RH level circularly ranges from 11% to 95% at 100 Hz. (**e**) The 100 cycle continuous response-recovery test for Pyr-rGO based humidity sensors measured at 100 Hz as RH level alternately changes between 11% and 95%.

It is generally known that the response of humidity sensors is dependent on the testing frequency which should be determined first before investigating the humidity sensing properties^29^. Hence, the impedance of Pyr-rGO sensors at different RH levels was tested with frequency ranged from 50 Hz to 100000 Hz. As can be seen from Fig. 3a, at almost all the frequencies, the impedance decreased along with the increase of RH level from 11% to 95%. Besides, to any RH level, the impedance decreased as the frequency rose from 50 Hz to 100000 Hz, indicating that the frequency influenced the impedance significantly in humidity sensing. However, the impedance curves were nearly linear at high frequencies of 1000 Hz, 10000 Hz and 100000 Hz, which were not desirable as the ideal testing frequency since the poor precision with extremely small response to humidity variation. For the remaining two frequencies, the curve of 100 Hz showed apparently wider span and more regular decrease on impedance towards humidity variation which revealed the higher sensitivity and accuracy than 50 Hz. Therefore, the optimum testing frequency for the following humidity sensing was set as 100 Hz.

For humidity sensors, the response, response-recovery time and repeatability are the key parameters to directly determine the accuracy and efficiency in practical test. Figure 3b and c showed the five-cycle response-recovery curve of Pyr-rGO and rGO measured at 100 Hz as RH level alternately changed between 11% and 95%. Herein, the response was calculated by the ratio of impedance captured at 11% RH (I_L_) and 95% RH (I_H_), respectively. By calculation (Fig. 3b), the Pyr-rGO sensors showed excellent humidity sensing performance with an ultrahigh response (I_L_/I_H_ = 6000) and an ultrafast response time (<2 s). In addition, the recovery time could be controlled within 6 s for such a high-expansion response of Pyr-rGO sensors. It was noteworthy that the Pyr-rGO sensors also exhibited remarkable repeatability, and the response remained highly stable (I_L_/I_H_ = 6000) with no attenuation in the impedance (I_L_ and I_H_) even after 100 cycle continuous response-recovery test (Fig. 3e). The Pyr-rGO based humidity sensor exhibited outstanding sensing performance compared to the reported ones summarized in Table 1. In contrast, the rGO sensors showed a low response (I_L_/I_H_ = 1.3) with a long response time for more than 50 s. Moreover, the repeatability of rGO sensors was unqualified that the response and impedance (I_L_ and I_H_) attenuated significantly after the five-cycle test as shown in Fig. 3c. To sum up, the Pyr-rGO based humidity sensors exhibit ultrafast and ultrahigh response (30 times faster and 4600 times higher than rGO sensors) with stable repeatability, indicating that the humidity sensing performance of Pyr-rGO has been prominently improved after the supramolecular assembly with pyranine.

**Table 1:** Comparison in sensing performance towards various humidity sensors.

Sensing Materials	Methods	RH Range	Response Time	Sensor Type^a^	Response
GO/SnOx/CF^38^	Electrospinning	30–55%	8 s	Resistance	3.35
rGO/PDAA^39^	LBL Self-assembly	11–97%	108 s	Resistance	1.6^b^
Graphene Oxide^40^	Drop-casting	15–95%	10.5 s	Capacitance	378
MoS_2_/SnO_2_^41^	Hydrothermal Method	0–97%	5 s	Capacitance	>10^5^
Na-Mesoporous Silica^42^	Hydrothermal Method	11–95%	47 s	Impedance	>10^5^
CuO/rGO^29^	Microwave-assisted Hydrothermal Method	11–98%	2 s	Impedance	22700^c^
WO_2.72_ Crystals^43^	Thermal Evaporation	11–95%	6 s	Impedance	3.77^b^
Pyr-rGO (*This work*)	Supramolecular Assembly	11–95%	<2 s	Impedance	6000

^a^Three widely adopted humidity sensor types are listed in this table, which records the change of resistance, capacitance and the impedance respectively in humidity testing. ^b^For the convenience of comparison, the evaluation of response is converted as the ratio of the resistance or impedance in high and low RH level. ^c^The testing frequency is 10 Hz while the other humidity sensors of impedance type are tested in 100 Hz.

In practical humidity sensing applications, the humidity hysteresis phenomenon is also an important parameter to evaluate the practicability of sensors, which is unavoidable but expected to be minimized. The reason for the existence of humidity hysteresis is that different energy is required during the humidification and dehumidification process. Larger desorption energy is always needed than adsorption energy, signifying the corresponding impedance cannot return to the adsorption impedance at the same time, and thus forms the humidity hysteresis. Figure 3d exhibits the humidity hysteresis curve of Pyr-rGO based humidity sensors. The hysteresis loops exhibited the impedance changes at 100 Hz during the stepwise successive humidification and dehumidification cyclical process measured from 11% RH to 95% RH. Notably, the loops were nearly overlapped at low or high RH levels and the dehumidification curve was slightly lower than the one in humidification. Moreover, the relative maximum hysteresis appeared at 54% RH was calculated with a small value of around 8% RH, indicating that the Pyr-rGO sensors are highly reversible with reliable practicability.

## Discussion

**Figure 4:**
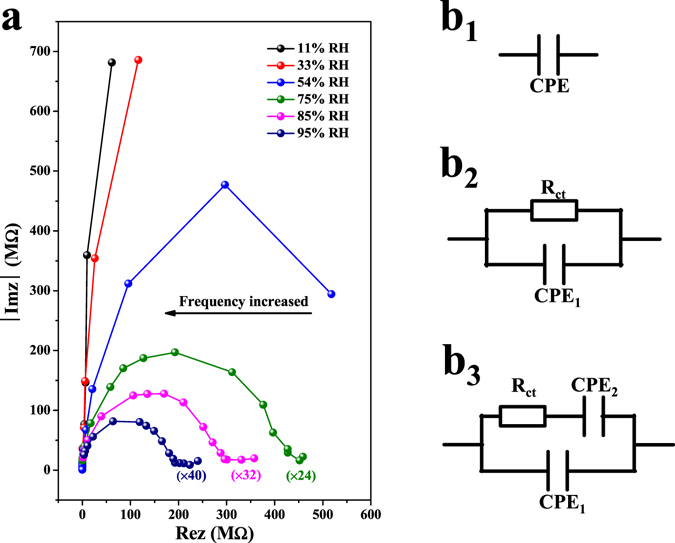
(**a**) The complex impedance spectra of Pyr-rGO based humidity sensors at different RH levels. ImZ: imaginary part; ReZ: real part. (**b**) The equivalent circuit at (b_1_) low (b_2_) middle and (b_3_) high RH range.

Complex impedance spectra (CIS) can be adopted to investigate the sensing mechanism of humidity sensors^30, 31^. Figure 4a showed the CIS of Pyr-rGO based humidity sensors as the RH value ranged from 11% to 95% under the operation frequency increased from 10 Hz to 100000 Hz at room-temperature. The real part and imaginary part of the CIS at high RHs were magnified on the same plane to compare the others more conveniently. Notably, the curve shape was quite different for diverse RH levels in CIS, suggesting that different humidity sensing mechanisms for water adsorption and ions transfer were adopted based on the RH values. Further, equivalent circuit (EC) models were built to clearly analyze the humidity sensing mechanism under different RH levels as shown in Fig. 4b. Here, R_ct_ represents the resistance of charge transfer, the constant phase element (CPE) stands for the sensing film capacitance (CPE_1_) and electrode/sensing film interface capacitance (CPE_2_).

At low RH range (11% and 33%), the CIS ran in almost straight lines and thus the EC model in this case could be described as a CPE (Fig. 4b_1_). As well-known, the impedance of a CPE is generally defined as Equation (1):^9, 32^

1 $${Z}_{CPE}=\frac{{(2\pi f)}^{-n}}{A}$$

2 $$\mathrm{log}|{Z}_{CPE}|=-n\,\mathrm{log}\,f+\,\mathrm{log}\,\frac{1}{{(2\pi )}^{n}A}$$

**Figure 5:**
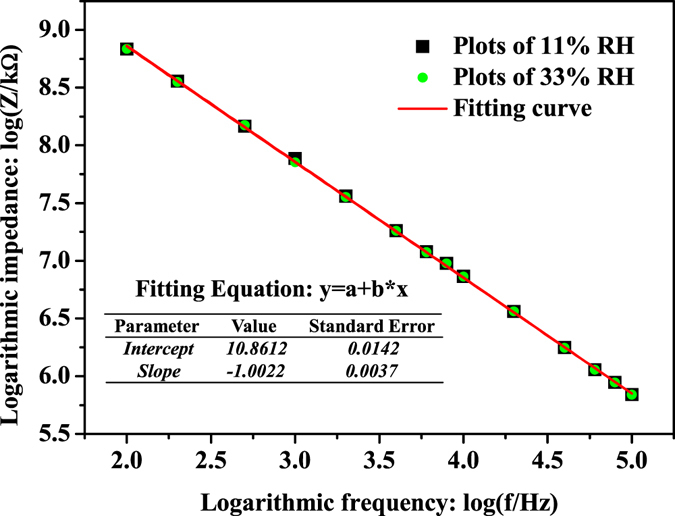
Linear fit plot of log Z vs. log f for Pyr-rGO at 11% and 33% RH.

Based on Equation (2), if we drew a plot of $\mathrm{log}|{Z}_{CPE}|vs\,\mathrm{log}\,f$, it was obvious that the plot should be a straight line with slope coefficient of −n. Actually the experimental plots of $\mathrm{log}|{Z}_{CPE}|vs\,\mathrm{log}\,f$ at 11% and 33% RH were indeed straight lines with negative slope coefficient of −1.0022 as shown in Fig. 5, which was highly in accordance with the theoretical analysis. At the low RH range, the chemical adsorption is the main adsorption method in humidity sensing, but only a few water molecules can be adsorbed onto the surface of Pyr-rGO film in this case. After the chemical adsorption, a proton maybe transferred to a water molecule to form H_3_O^+^, however, the proton can only migrate by hopping from site to site across the Pyr-rGO film surface^33^. Therefore, the impedance is relatively high at low RH range since the weak ion transport on account of the uncontinuous and low water coverage.

At middle RH (54%), the curve of CIS was part of semicircle, which was mainly generated from the intrinsic impedance of the sensing film. It could be described by a simplified EC model comprising a resister and a capacitor connected in parallel^34, 35^, as displayed in Fig. 4b_2_. The appearance of a semicircle in CIS suggested the existence of H^+^ hopping conduction^33^. According to the ion transfer mechanism of Grotthuss, H_2_O + H_3_O^+^ = H_3_O^+^ + H_2_O^36^, the initial and final state are the same and the energies are also balanced, thus the ion transfer is quite easy. Moreover, at this RH level, more water molecules can be adsorbed onto Pyr-rGO film due to the hydrophilic sulfonic groups of pyranine, as reported in our previous work^18^. The absorbed water molecules can form one or several serial water layers which accelerate the transfer of H^+^ or H_3_O^+^, resulting in a swift decrease in the impedance at this stage.

At high RH range (75%, 85% and 95%), the CIS exhibited a semicircle shape with a short straight line at low frequency region. As the RH increased, the straight line grew longer while part of the semicircle disappeared. Actually, the short straight line represented Warburg impedance, caused by the ions diffusion at sensing material/electrode interface^37^. Therefore, the EC model shown in Fig. 4b_3_ was suitable for the sensing mechanism of Pyr-rGO at this stage. At high RH range, apart from the chemical adsorption, the physical adsorption has upgraded as the main adsorption method. Thus, more serial water layers are formed to further accelerate the ions transfer, and the water molecules can permeate into the Pyr-rGO film to induce the electrolytic conduction. Therefore, the impedance continuously decreases sharply at high RH range.

In summary, the Pyr-rGO based humidity sensing materials were prepared through a facile one-step supramolecular assembly method. The modification of rGO by organic molecule, *i.e*. pyranine, realized the synergetic functionalization to enhance the dispersibility and humidity sensing properties. The fabricated Pyr-rGO humidity sensors exhibited comprehensive sensing performance such as ultrafast response (<2 s) and ultrahigh response (I_L_/I_H_ = 6000) as RH alternately changed between 11% and 95%; small hysteresis of 8% RH; reliable repeatability and stability. Further, the complex impedance spectra under different RH was discussed and the corresponding equivalent circuits were built to investigate the humidity sensing mechanism in detail, verifying that different water molecules adsorption and ion transfer were adopted in different RH levels. Notably, we believe that the Pyr-rGO represents a new platform for developing novel functional materials in ultra-sensitive humidity sensors and shows the potential for facile one-step humidity sensing materials preparation.

## Methods

### Materials

GO flakes were obtained from XianFeng NANO Co., Ltd. Pyranine (*i.e*. Trisodium 8-hydroxypyrene-1,3,6-trisulphonate, >85%) and hydrazine hydrate (>98%) were purchased from Alfa Aesar. Ammonium hydroxide (28% NH_3_ in H_2_O), lithium chloride (≥99%), magnesium chloride (99.99%), magnesium nitrate (≥99%), sodium chloride (≥99%), potassium nitrate (≥99%), and potassium chloride (≥99%) were all purchased from Sigma-Aldrich. All the chemicals were analytical grade and used as received without further purifications.

### Preparation of rGO Dispersion

GO flakes were dispersed into deionized (DI) water under vigorous ultra-sonication for an hour to prepare a brown GO dispersion of 1 mg/mL. 5 mL GO dispersion (1 mg/mL) was transferred into a 50 mL round-bottom flask and diluted by 15 ml DI water. Subsequently, 80 μL ammonium hydroxide (30%) and 10 mL hydrazine hydrate (0.1%) were added respectively and heated at 95 °C for 1 h in oil bath. After cooling to ambient temperature, the mixture was filtrated to dry and then re-dispersed into 20 mL DI water under mild sonication for 5 min to prepare 0.25 mg/mL rGO dispersion.

### One-Step Preparation of Pyr-rGO Dispersion

5 mL GO dispersion (1 mg/mL) was transferred into a 50 mL round-bottom flask and diluted by 15 ml DI water. Pyranine (100 mg), ammonium hydroxide(80 μL, 30%) and hydrazine hydrate (10 mL, 0.1%) were added into the dispersion respectively under mild stir for 30 mins and then the mixture was further stirred at 95 °C for 1 h in oil bath. After cooling to ambient temperature, the dispersion was transferred to a 50 mL centrifuge tube and washed by DI water under 10 mins centrifugation at 12000 r/min for three times. The residuum was then re-dispersed into 20 mL DI water to prepare 0.25 mg/mL Pyr-rGO dispersion.

### Fabrication of Humidity Sensors

**Figure 6:**
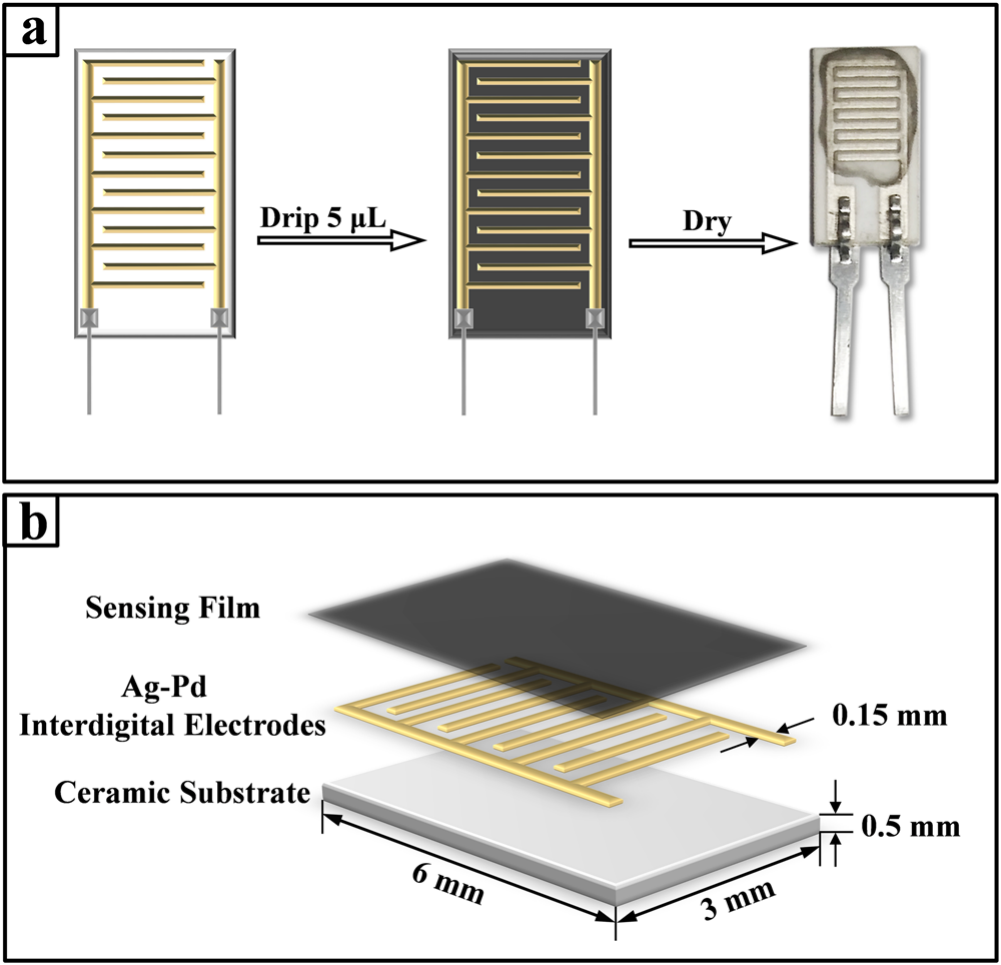
(**a**) The schematic diagram for fabricating humidity sensor by Drop & Dry method and typical optical image of a fabricated humidity sensor. (**b**) The component and overall dimensions of the humidity sensor.

In the fabrication of humidity sensors, a facile Drop & Dry method was applied by dropping approximately 5 μL Pyr-rGO dispersion on Ag-Pd Interdigital electrodes (IEs) and then dried on heating plate in air at 50 °C for 10 min. Finally, a thin sensing film was formed on the surface of IEs and thus the humidity sensors were prepared for testing as shown in Fig. 6a. For the convenience to connect humidity sensing system, two iron wires were anchored at the bottom of IEs by tin soldering. The sensors were made of ceramic substrates (6 mm × 3 mm × 0.5 mm) with five pairs of Ag-Pd IEs (0.15 mm in wire width) fixed on them as shown in Fig. 6b.

### Humidity Sensing Tests

All the humidity sensing tests were performed by a humidity sensing system (CHS-1 Humidity Sensing Analysis System, Beijing Elite Tech. Co.) at an operating temperature of 25 °C. The voltage in humidity test was 1 V AC, and the testing frequency varied from 50 Hz to 100000 Hz. The humidity gradient was controlled by several erlenmeyer flasks filled with different saturated salt solutions which were LiCl, MgCl_2_, Mg(NO_3_)_2_, NaCl, KCl, and KNO_3_ with corresponding relative humidity (RH) of 11%, 33%, 54%, 75%, 85%, and 95% respectively. To ensure the accuracy of RH, 10 h stabilization of the flasks was necessary to get fully liquid-gas equilibrium before humidity sensing test and the ambient RH was maintained at 25% by an automatic drier. The ideal flow chart of the humidity sensing test was indicated in Supplementary Figure S3.

### Characterization

The humidity sensing materials were characterized by several techniques. The morphology was characterized by transmission electron microscope (TEM) (Sirion-200, Japan). The surface properties were examined by FT-IR (Thermo Scientific Nicolet iN10, USA), Raman-scattering spectroscopy (HORIBA Jobin Yvon Raman microscope (LabRAM HR800) and X-ray photoelectron spectra (XPS; ESCALAB 250 photoelectron spectrometer (ThermoFisher Scientific, USA).

## Electronic supplementary material

Supplementary information
