# Supplementary material for: One-Step Fabrication of Pyranine Modified- Reduced Graphene Oxide with Ultrafast and Ultrahigh Humidity Response
Source: Sci Rep. 2017 Jun 2;7:2713. doi: 10.1038/s41598-017-02983-8 (PMC5457436; doi:10.1038/s41598-017-02983-8)
Supplement: Supplementary file 1 — Supplementary information [file 41598_2017_2983_MOESM1_ESM.pdf]

Supporting Information

One-Step Fabrication of Pyranine Modified-Reduced Graphene Oxide with Ultrafast and Ultrahigh Humidity Response

Zhuo Chen,[1] Yao Wang,[1,*] Ying Shang,[1] Ahmad Umar,[2] Peng Xie,[1] Qi Qi[3] and Guofu Zhou[4,*]

[1]*Key Laboratory of Bio-Inspired Smart Interfacial Science and Technology of Ministry of Education, School of Chemistry and Environment, Beihang University, Beijing 100191, P. R. China.*

[2]*Department of Chemistry, Faculty of Science and Arts and Promising Centre for Sensors and Electronic Devices (PCSED), Najran University, Najran 11001, Kingdom of Saudi Arabia.*

[3]*Gas and Humidity Sensing Department, Beijing Elite Tech Co., Beijing 100850, PR China.*

[4]*Institute of Electronic Paper Displays, South China Academy of Advanced Optoelectronics, South China Normal University, Guangzhou 510006, P. R. China.*

***Correspondence and requests for materials should be addressed to:**

Y. W. (email: yao@buaa.edu.cn) or G. Z. (email: guofu.zhou@m.scnu.edu.cn)

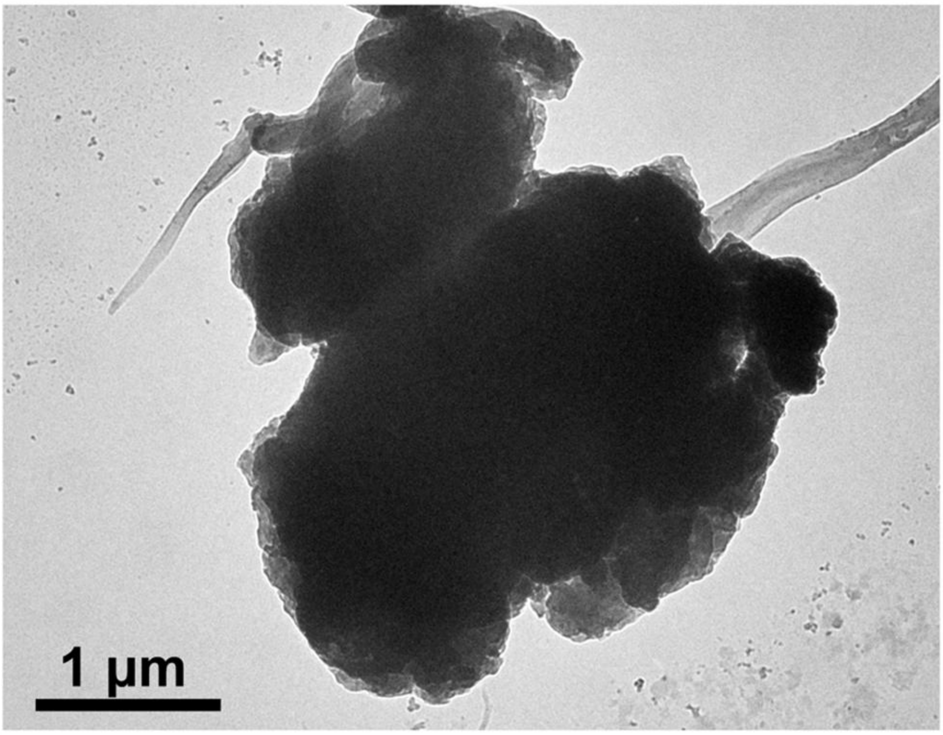

Figure S1. The TEM image of stacked rGO sheets.

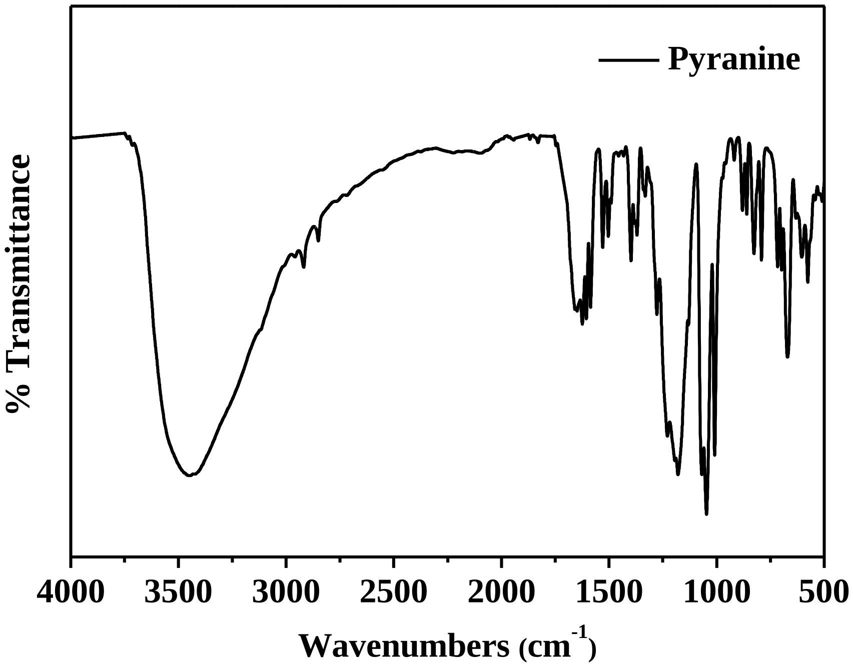

Figure S2. The spectra of pure pyranine powders.

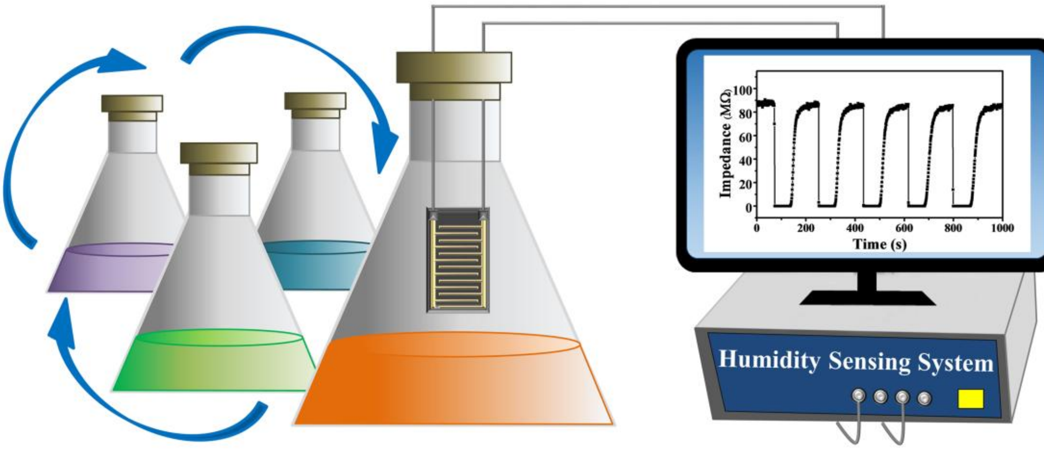

Figure S3. An ideal flow chart of the humidity sensing test.

Table S1. The XPS peak table of Pyr-rGO.

Name	Start BE	Peak BE	End BE	PP At. %
**C1s**	297.5	284.81	279.1	63.98
**O1s**	547.4	532.28	525.5	20.07
**S2p**	180.5	168.58	163.94	1.83
**Ca2p**	361.5	347.47	341.6	1.03
**N1s**	418.5	399.88	388.6	3.72
**Na1s**	1083.5	1071.1	1066.6	2.52
**Si2p**	114	101.97	96.1	6.85

The calculated C/S atomic ratio is 34.96, indicating that each pyranine molecule was assembled with 89 carbon atoms of graphene. Approximately, the coverage of pyranine on the graphene is around 18% based on a rough calculation.
